# P-2096. Prevalence of Sexually Transmitted Infections Among Female Adolescent Victims of Sexual Violence in Korea

**DOI:** 10.1093/ofid/ofaf695.2260

**Published:** 2026-01-11

**Authors:** Soo-Han Choi, Su Eun Park

**Affiliations:** Pusan National University School of Medicine, Busan, Republic of Korea; Pusnal National University School of Medicine, Busan, Pusan-jikhalsi, Republic of Korea

## Abstract

**Background:**

Adolescents are known to be at higher risk for sexually transmitted infections (STIs) due to behavioral and biological factors. Compared to adults, STI diagnosis and treatment are often delayed in this population. STIs can cause a range of health problems in adolescents, yet data on STIs among Korean adolescents are limited.

**Methods:**

We retrospectively reviewed female adolescents aged ≤18 years who underwent STI testing at Pusan National University Hospital via a Sunflower Center, an integrated service center for victims of violence in South Korea, from July 2017 to February 2024. STI was defined as a positive result for HIV antibody or VDRL in blood tests or detection of *Chlamydia trachomatis*, *Neisseria gonorrhoeae*, *Treponema pallidum*, *Mycoplasma genitalium*, *Trichomonas vaginalis*, HSV-1, or HSV-2 via multiplex PCR on cervical swabs.

**Results:**

A total of 123 cases were reviewed. The median age was 15.5 years (range: 11.5–18.9), with 12.2% aged ≤12 years, and 43.9% aged 13–15 or 16–18 years, respectively. Of these, 58.5% reported prior sexual experience, and 9.8% had unclear histories. The overall positive rate for STI testing was 35.0% (43/123), with rates of 20.0% in those ≤12 years, 40.7% in ages 13–15, and 33.3% in ages 16–18. Among those with prior or unclear sexual experience, the positive rate for STI testing was 41.7%, compared to 20.5% among those without sexual experience. The most frequently detected pathogen was *C. trachomatis* (29.3%), followed by *M. genitalium* (12.2%), *N. gonorrhoeae* and HSV-2 (each 4.1%). Multiple pathogens were detected in 37.2% of STI-positive cases. Additionally, 92.7% of all cases tested positive for at least one of *Mycoplasma hominis*, *Ureaplasma* species, *Gardnerella vaginalis*, or *Candida albicans*, with 97.7% co-detection in STI-positive cases.

**Conclusion:**

Understanding the risk of STI exposure among adolescents is critical for effective prevention and timely treatment of STIs in adolescents.

**Disclosures:**

All Authors: No reported disclosures

